# Antimicrobial and Hemostatic Diatom Biosilica Composite Sponge

**DOI:** 10.3390/antibiotics13080714

**Published:** 2024-07-30

**Authors:** Sol Youn, Mi-Ran Ki, Ki Ha Min, Mohamed A. A. Abdelhamid, Seung Pil Pack

**Affiliations:** 1 Department of Biotechnology and Bioinformatics, Korea University, Sejong-Ro 2511, Sejong 30019, Republic of Korea; youseul0419@korea.ac.kr (S.Y.); allheart@korea.ac.kr (M.-R.K.); alsrlgk@korea.ac.kr (K.H.M.); mohamed42@korea.ac.kr (M.A.A.A.); 2 Institute of Industrial Technology, Korea University, Sejong-Ro 2511, Sejong 30019, Republic of Korea; 3 Department of Botany and Microbiology, Faculty of Science, Minia University, Minia 61519, Egypt

**Keywords:** diatom biosilica, doxycycline, drug delivery, wound sponge, gelatin, tooth extractions

## Abstract

The 3D nanopatterned silica shells of diatoms have gained attention as drug delivery vehicles because of their high porosity, extensive surface area, and compatibility with living organisms. Tooth extraction may result in various complications, including impaired blood clotting, desiccation of the root canal, and infection. Therapeutic sponges that possess multiple properties, such as the ability to stop bleeding and kill bacteria, provide numerous advantages for the healing of the area where a tooth has been removed. This study involved the fabrication of a composite material with antibacterial and hemostatic properties for dental extraction sponges. We achieved this by utilizing the porous nature and hemostatic capabilities of diatom biosilica. The antibiotic used was doxycycline. The gelatin-based diatom biosilica composite with antibiotics had the ability to prevent bleeding and release the antibiotic over a longer time compared to gelatin sponge. These properties indicate its potential as a highly promising medical device for facilitating rapid healing following tooth extraction.

## 1. Introduction

Diatoms, unicellular photosynthetic algae with 3D nanopatterned silica shells known as frustules, can be utilized as efficient drug carriers in clinical environments. Frustule is characterized by its high porosity, large specific surface area, biocompatibility, thermal stability, and adjustable surface chemistry. These qualities make diatoms well-suited for drug delivery purposes [1,2]. Furthermore, diatoms can be economically obtained from plentiful, fossilized sediments known as diatomaceous earth. This offers a cost-effective substitute for synthetic porous silica [3]. Diatoms can serve as carriers for various therapeutic substances, such as chemotherapeutic drugs, siRNAs, and gene therapies [4,5]. They also have important uses in biosensing and imaging, which play a crucial role in cancer diagnosis and treatment [6]. Additionally, their environmentally friendly characteristics and capacity to be cultivated in regulated settings enhance their sustainability and mitigate environmental pollution problems linked to chemical production [7]. Vasani et al. developed a microcapsule from diatom biosilica that can release drugs, such as the antibiotic levofloxacin, in response to temperature changes, which is useful for controlled drug delivery systems [8]. Liu et al. demonstrated that a new composite material made from diatomaceous earth and zinc oxide (DE-ZnO) enhanced the effectiveness of antibiotics against fungi and Gram-negative bacteria [9]. Combining DE-ZnO with existing antifungal drugs like itraconazole and amphotericin B results in better antifungal activity, which can reduce the side effects associated with high doses of these drugs.

Tooth extraction is a dental procedure in which the tooth is completely removed from the alveolus [10]. This procedure is performed when the teeth are severely damaged beyond the point of treatment, such as wisdom tooth extraction, severe cavities, fractured teeth, impacted teeth, severe gum disease, tooth dislocation, or dental injuries [11,12,13]. Typically, after extraction, gauze is placed over the extraction site, and pressure is applied to slow bleeding [14]. However, a simple gauze does not allow blood to coagulate properly or has no antibacterial effect. As these disadvantages can lead to complications such as infection, dry socket, dry cavities, and nerve injury, new strategies to replace gauze after tooth extraction are needed to prevent them [15,16].

Sponges made using gelatin are a new alternative. Gelatin has attracted attention for many uses in the medical field owing to its high biocompatibility, low cost, and wide availability [17,18]. Additionally, through the crosslinking process, it can be shaped into a sponge that can enter the tooth extraction area. However, it is important not only to establish a shape to prevent complications but also to improve hemostasis, antibacterial properties, compressive strength, biocompatibility, and bone regeneration. Diatom biosilica, also known as diatomaceous earth or diatomite, possesses porous properties that enable the delayed release of drugs immersed within it. In addition, this porous structure is also responsible for its hemostatic effect when applied to wounds [19]. The porous architecture with hydrophilicity absorbs blood quickly into the diatom frustule, allowing silica and blood to come into contact immediately. The porous silica surface and blood interact to activate coagulation factor XII (FXII), which is essential for blood clotting [19]. Therefore, a sponge composite made of gelatin and diatom biosilica can be expected to have a hemostatic effect from diatom silica. Hemostasis not only stops the loss of blood after a wound, but the components involved in hemostasis, such as the coagulation system, platelets, and the vascular system, also play significant roles in other processes like wound healing and angiogenesis [20]. Therefore, they help wounds heal as the first step in wound repair [21]. Additionally, the combined application of antibiotics ensures that the sponge has antibacterial properties and allows the wound to heal easily, without requiring additional treatment. Moreover, owing to the delayed release ability of diatomite, antibiotics are continuously released and stably delivered to extraction sites [22].

Doxycycline is a tetracycline-class antibiotic that effectively fights against various infections, such as anthrax, Lyme disease, cholera, and syphilis [23]. Doxycycline inhibits bacterial protein biosynthesis, thereby exerting its antibacterial activity [24]. Doxycycline has been utilized in various dental procedures, such as the management of periodontal disease and peri-implantitis [25]. Doxycycline hyclate has been shown to be effective in the treatment of periodontal disease in adults. It is known to inhibit matrix metalloproteinases that contribute to the pathological degradation of connective tissue collagen in the periodontal supporting structures within the gingival crevicular fluid [26]. Moreover, research has shown that doxycycline can promote bone formation markers [25] and stimulate a significant rise in active osteoblasts [27]. Therefore, doxycycline can be a useful medication for maintaining the health of periodontal tissue, treating bacterial infection, inflammation, and promoting bone formation in various dental procedures, including tooth extraction, periodontitis treatment, and dental implant procedures [26,28,29,30].

Researchers have developed and analyzed doxycycline-loaded microspheres using biodegradable polymers [23,31,32,33]. In this study, diatom biosilica was selected as a drug delivery carrier for doxycycline due to its porous structure and hydrophilic surfaces, which enable it to function not only as a drug carrier but also to promote blood adsorption and coagulation [19]. The goal was to assess the effectiveness of diatom biosilica as a drug carrier by evaluating its antibacterial activity against microorganisms after drug immersion, and to explore its potential application in dental treatments following tooth extraction in the form of a gelatin-based composite sponge.

## 2. Results and Discussion

### 2.1. Comparison of the Antimicrobial Activity of Antibiotics before and after Soaking in Diatom Biosilica

**Figure 1:**
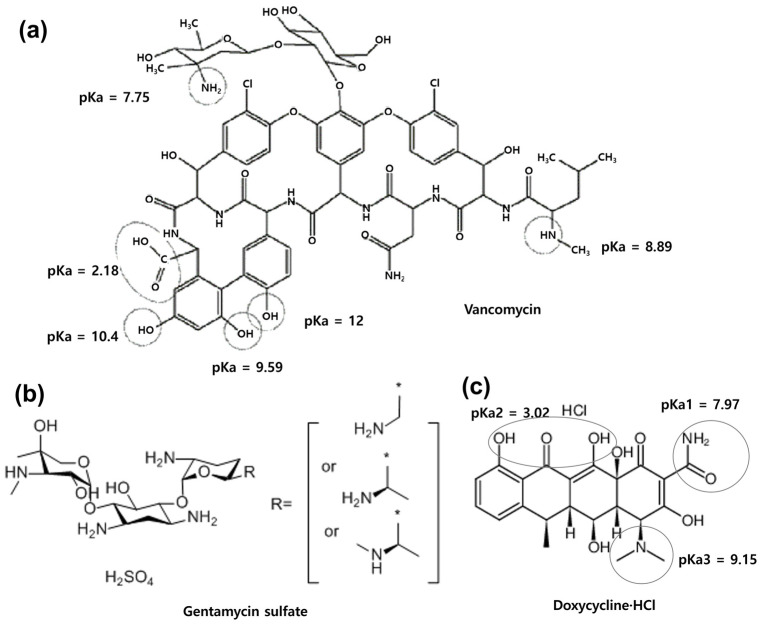
Structures of antibiotics used. (**a**) Vancomycin (VM). Reproduced with permission from [40]. Copyright 2006 Springer Nature. (**b**) Gentamycin sulfate (GEN). An asterisk denotes the remainder of the gentamycin structure, excluding the R group. Adapted from [41], © Copyright DRUGBANK online, 2024. (**c**) Doxycycline·HCl (DC). Adapted from [42], © Copyright DRUGBANK online, 2024. The p*K*a values are taken from Shariati et al. [43].

To assess diatom biosilica (DB) as an antibiotic delivery carrier, vancomycin (VM), gentamycin sulfate (GEN), and doxycycline hydrochloride (DC) were employed. The characteristics of the antibiotics used are summarized in Supplementary Table S1 and the structures of each antibiotic are shown in Figure 1. The adsorption rate of each antibiotic in DB was examined. VM is a glycopeptide antibiotic utilized for the treatment of severe gram-positive bacterial infections [34]. It falls under the category of antibacterial drugs known as cell wall synthesis inhibitors. GEN is an aminoglycoside antibiotic used to treat various types of bacterial infections. It exhibits efficacy against a wide range of gram-negative bacteria and certain gram-positive bacteria [35]. It functions by attaching to the bacterial ribosome, impeding protein synthesis, and ultimately causing the demise of the bacteria [36]. DC is a tetracycline antibiotic that is soluble in water. It has the ability to kill and inhibit the growth of a broad spectrum of bacteria, both gram-positive and gram-negative [37]. Multiple studies have demonstrated that tetracyclines, specifically doxycycline, possess immunomodulatory properties and can effectively manage inflammation in conditions like rheumatoid arthritis [38]. It has also been reported to limit amyloid-like aggregation of α-synuclein and reduce inflammatory processes associated with neurodegeneration [39].

The loading efficiency of the antibiotics in DB, calculated as the adsorption into 1 mg DB per 300 μg of each antibiotic, is presented in Table 1. As a control for the porous DB, a 4 μm size solid silica particle (SP) without porosity was used to immerse VMs. In the SP system, the adsorbed rate of VMs was approximately 18% of the initially injected amount. However, in the DB system, the adsorbed rate of antibiotics reached 60%, which is more than three times that of the SP system. The adsorption efficiency onto DB followed the order DC > GEN > VM. Both GEN and DC demonstrated high adsorption rates, with more than 90% of the added amount being adsorbed by the prepared DBs. The loading efficiencies (LE) for the antibiotic DB carriers were 15.26% for VM, 21.38% for GEN, and 21.70% for DC. Based on the physiological charges presented in Table S1, VM, with a physiological charge of zero, exhibited a lower adsorption rate compared to GEN, which has amine groups and a physiological charge of 5 (Supplementary Table S1 and Figure 1). DB exhibits negatively charged surfaces, based on its zeta potential value (−30.83 ± 0.23 mV) as shown in Supplementary Table S2. Consequently, GEN has a stronger interaction with DB than VM does due to electrostatic attraction, resulting in higher LE and AE. Given that the VM is devoid of a physiological charge, whereas the carboxyl group of it exhibits a negative charge at physiological conditions such as phosphate buffered saline solution (PBS) (Figure 1a), electrostatic repulsion may ensue during the adsorption process with the adsorption sites of the DB, which could be disadvantageous for the access of the VM to the DB. Meanwhile, the high adsorption rate of DC does not appear to be attributable to electrostatic attraction with DB, given that DC has a physiological charge of zero. However, an examination of the structure of DC (Figure 1c) and p*K*a3 suggests that the ammonium represents a cation in PBS, thereby enabling binding with the anion of DB by electrostatic attraction [44]. In addition, its threefold smaller size in comparison to VM may have contributed to the increased adsorption rate. After the adsorption of each antibiotic by the DB, there was a noticeable change in the zeta potential (Supplementary Table S2). The incorporation of GEN resulted in a shift from approximately −30 to approximately 10 mV, attributed to the influence of the cationic GEN bound to the surface. Additionally, the adsorption of VM and DC led to a slight increase in anionicity, measuring −33 and −36 mV, respectively (Supplementary Table S2). When the ammonium group of DC binds to the anion of DB, it moves the positively charged part of DC toward the binding site of DB [45]. This pulls the negatively charged part of DC to the surface of DB. As a result, DC@DB exhibits higher electronegativity than DB. It is believed that VM@DB has higher electronegativity values for similar reasons to DC.

The antibacterial activity of antibiotics was then investigated before and after immersion in DB against Gram-negative *Escherichia coli* (*E. coli*) and Gram-positive *Staphylococcus aureus* (*S. aureus*). The antibacterial activity of antibiotics and antibiotic-loaded DBs was compared using the minimum inhibitory concentration (MIC) measurement (Table 2). VM demonstrated lower antimicrobial efficacy against *E. coli*, with MIC values of 115.22 ± 51.53 μgmL^−1^. Conversely, VM showed superior antimicrobial activity against *S. aureus*, with a MIC of 3.75 ± 1.26 μgmL^−1^. Interestingly, VM loaded in DB exhibited a lower MIC (56.25 ± 15.73) against *E. coli* and a similar MIC (4.89 ± 2.05) against *S. aureus* as the free form. Both GEN and DC demonstrated superior antimicrobial activity compared to VM against both strains (Table 2). When comparing the antibacterial activity of the antibiotics in their free form versus when loaded in DB, it was found that GEN exhibited a sevenfold higher MIC against *E. coli* and a fourfold higher MIC against *S. aureus*, while DC exhibited approximately a fivefold higher MIC against *E. coli* and a threefold higher MIC against *S. aureus*. VM exhibited comparable or enhanced activity, while GEN and DC showed reduced activity. Specifically, the low antimicrobial activity of the VM against *E. coli* was increased when loaded into the DB, suggesting that the DB enhanced the delivery of the VM into *E. coli*. The similar antibacterial activity of the free form against *S. aureus* was attributed to the low binding strength of VM to DB, which facilitated its release after binding to DB. However, in the case of GEN and DC, the antibacterial potency was reduced compared to the free form, indicating that either the GEN or DC bound to DB exhibits sustained release due to the strong binding force with DB.

A comparative analysis of three kinds of antibiotics demonstrated the potential of DB as a carrier for a range of antibiotics. The chemical characteristics of drugs are of significant importance with regard to their binding to DB and the subsequent antibiotic effect that ensues following loading into DB. Most notably, the porous structure facilitates the adsorption of diverse antibiotics through straightforward immersion. In the case of patients with drug allergies or individual sensitivity to the drug, it is of the utmost importance that they utilize a medication that aligns with their presenting symptoms [46]. DB’s high adsorption capacity for a range of drugs enables medical advisors to select alternative drugs that suit the patient’s needs and then load the drug into the DB on the spot through a simple soak rather than a complex formulation. However, strongly cationic drugs may experience difficulties with release after loading into DB [47], making the application of drugs with neutral physiological conditions and cationic functional groups more beneficial.

Our next objective is to explore its potential as a practical material. We investigated the feasibility of using DB to create a composite material with hemostatic, antibacterial, and anti-inflammatory properties for post-tooth extraction use. For this, DC has been selected as an antibiotic to be loaded into DB, as it is used for dental inflammation and infection as well as beneficial effects on bone formation [25,27,48].

### 2.2. Anti-Inflammatory Effect of DC and DC@DB on Lipopolysaccharide (LPS)-Induced Inflammation

**Figure 2:**
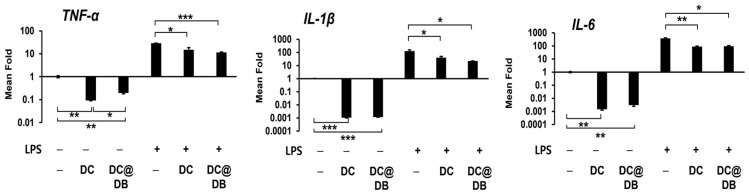
The anti-inflammatory response of DC and DC@DB in RAW264.7 cells was evaluated after stimulation with LPS. The mRNA expression levels of TNF-α, IL-1β, and IL-6 were analyzed using qRT-PCR, with normalization to the glyceraldehyde-3-phosphate dehydrogenase (GAPDH) gene. The gene levels in each treatment group are expressed as the mean fold change in expression compared to the group without any additives. The data is presented as the mean ± SE (N = 3). Statistical significance is indicated as * *p* < 0.05, ** *p* < 0.01, and *** *p* < 0.001.

To assess the ability of DC and DC@DB to neutralize LPS, we analyzed the mRNA expression levels of LPS-stimulated tumor necrosis factor alpha (TNF-α), interleukin 1 beta (IL−1β), and interleukin 6 (IL-6) in RAW264.7 macrophages (Figure 2). Following LPS treatment, the expression of these cytokines increased, but was significantly reduced by DC and DC@DB treatment, showing a decrease of approximately 46% and 57%, respectively. Moreover, the LPS-induced expression of *IL-1β* was reduced by approximately 70% and 80% with DC and DC@DB treatments, respectively. Similarly, the LPS-induced expression of *IL6* was reduced by approximately 75% with each treatment. Furthermore, the expression of these inflammatory cytokines was also reduced by DCs in cells without LPS induction. Additionally, DB alone increased the expression of proinflammatory cytokines, but suppressed the excessive expression induced by LPS (Supplementary Figure S1). This indicates that DB possesses immunomodulatory capabilities and could further reduce LPS-induced inflammatory responses in the presence of DC. While the anti-inflammatory activity of DC@DB showed an increasing trend compared to DC, it was not statistically significant. Apart from its antimicrobial properties, the immunomodulatory ability of DC@DB in infected tissues is believed to have a significant impact on healing by restoring the host’s protective immunity [38].

### 2.3. Effects of DC and DC@DB on TNF-α Exposed Osteoblasts

**Figure 3:**
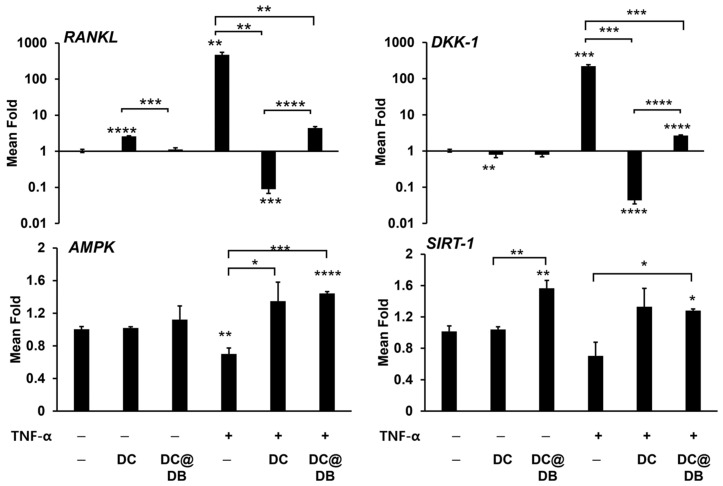
The anti-inflammatory response of DCs and DC@DBs in MC3T3 E1 cells was evaluated after stimulation with TNF-α. The mRNA expression levels of RANKL, DKK-1, AMPK, and SIRT-1 were analyzed using qRT-PCR, with normalization to GAPDH expression levels. The gene levels in each treatment group are expressed as the mean fold change in expression compared to the group without any additives. The data are presented as the mean ± SE (N = 3). Statistical significance is indicated as * *p* < 0.05, ** *p* < 0.01, *** *p* < 0.001, and **** *p* < 0.0001 vs. the control group without any additive. Comparisons between the two groups are indicated by brackets and asterisks for significance.

We investigated the impact of anti-inflammatory DC and DC@DB on the expression of RANKL, which promotes osteoclast differentiation, and DKK-1, which inhibits osteogenic differentiation, in osteoblasts exposed to the inflammatory cytokine TNF-α [49,50]. As shown in Figure 3, TNF-α treatment significantly increased the expression of *RANKL* and *DKK-1* in osteoblasts, while the presence of DC and DC@DB lowered their expression. Specifically, DC inhibited the expression of both more strongly than DC@DB and even more than TNF-α-unexposed control.

AMP-activated protein kinase (AMPK) serves as a crucial energy sensor and regulator of cellular metabolism [51]. It plays a significant role in preserving cellular energy balance and may facilitate protective responses to stress [52]. Sirtuins are a highly conserved family of NAD+-dependent deacetylases that serve as cellular sensors, detecting energy availability and regulating metabolic processes [53]. Mammalian Sirtuin 1 (SIRT-1) is responsible for deacetylating a variety of target proteins that play crucial roles in apoptosis, cell cycle regulation, circadian rhythms, mitochondrial function, and metabolism [53]. Overexpressing SIRT-1 has been demonstrated to effectively prevent cytokine-induced cellular damage [54] and reduce inflammatory responses [55]. SIRT-1 is a protein involved in regulating inflammation with antioxidant and anti-inflammatory properties. For example, SIRT-1 can reduce histone H3K9 acetylation at the promoters of IL-6 and TNF-α, thereby inhibiting their expression [56].

The expression of *AMPK* was found to decrease following TNF-α treatment, but this decrease was reversed in the presence of DC and DC@DB (Figure 3). *SIRT-1* expression was also reduced by TNF-α treatment but restored by DC and DC@DB. Both the increase in *AMPK* and *SIRT-1* by DC and DC@DB indicate potential cytoprotective effects through anti-inflammatory actions.

Next, we fabricated the gelatin-DB-DC composite material for the application of hemostatic sponges for dental care such as post-tooth extraction and investigated the feasibility of this composite.

### 2.4. Attenuated Total Reflection Fourier-Transform Infrared Spectroscopy (ATR−FTIR) of Gelatin-DB-DC Composites

**Figure 4:**
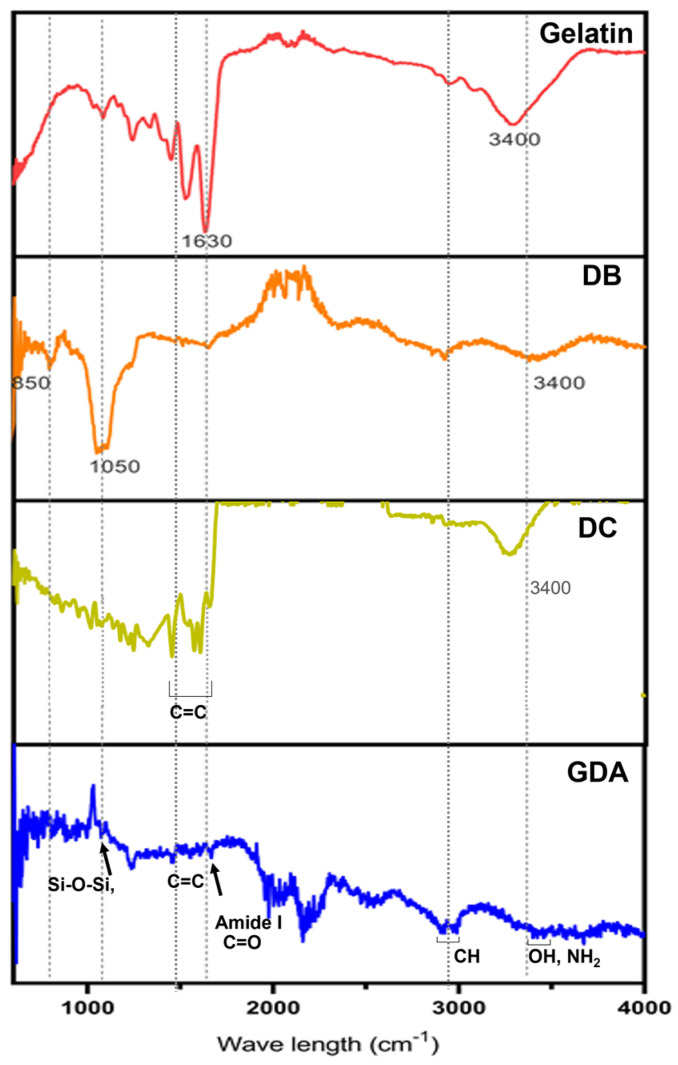
ATR−FTIR spectra of gelatin, DB, DC, and GDA.

Sponges made from gelatin sponge cross-linked using glutaraldehyde (G), sponge G dipped in antibiotic DC (GA), gelatin mixed with DB and cross-linked (GD), and sponge GD dipped in antibiotic DC (GDA) were prepared. Their composition was confirmed using ATR−FTIR. ATR−FTIR spectroscopy is a powerful analytical technique used to investigate the molecular composition of materials [57]. ATR−FTIR combines the principles of infrared spectroscopy with the ATR sampling method to provide valuable insights into the chemical bonds and functional groups present in a sample [57]. The ATR−FTIR spectra of composites are shown in Figure 4. The characteristic peaks of gelatin were observed at 3400 cm^−1^ and 1630 cm^−1^. These peaks correspond to the stretching vibration of the hydroxyl (OH) and amide (N-H) groups of gelatins, as well as the C=O stretching vibration, which is a distinctive peak indicative of the gelatin protein structure [58]. In DB, the O-H stretching vibration at 3400 cm^−1^, the Si-O stretching vibration at 1050 cm^−1^, and the Si-O-Si bending vibration at 850 cm^−1^ are observed [58]. The DC spectrum displays prominent peaks at 1500–1600 cm⁻^1^ (aromatic C=C stretching), 1666 cm⁻^1^ (C=O stretching), and 3250–3500 cm⁻^1^ (OH stretching) [33]. The major peaks observed in the GDA sponge samples indicate that the components, including gelatin and DB, were successfully incorporated into the GDA sponge and that the chemical structures of each component were determined.

### 2.5. Hemolysis and Hemostatic Ability

**Figure 5:**
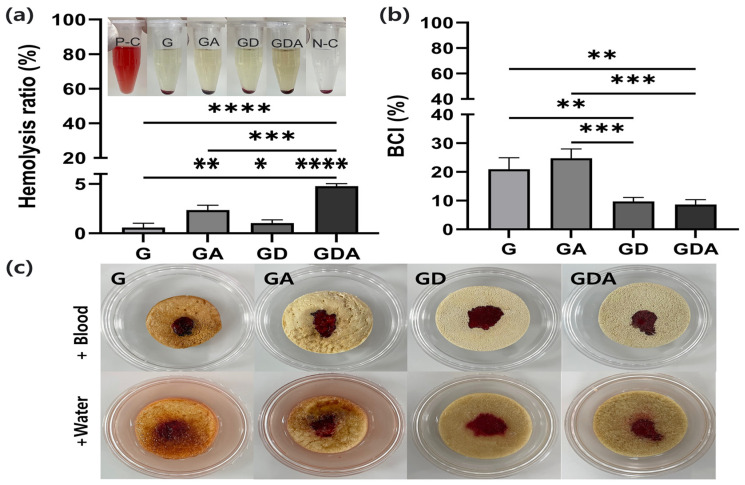
Hemolysis and hemostatic ability of sponge samples. (**a**) Hemolysis rates of the G, GA, GD, and GDA sponges. A higher activity indicates lower values. (**b**) BCI values of the G, GA, GD, and GDA sponges. A higher hemostatic ability indicates lower values. (**c**) Photographs of the G, GA, GD, and GDA sponges during blood hemostasis. * *p* < 0.05, ** *p* < 0.01, *** *p* < 0.001, and **** *p* < 0.0001.

Hemolysis is the rupture of a red blood cell (RBC) and the release of its contents. It is commonly used to measure the cytotoxic effect of a substance or condition. It is an important parameter for evaluating the safety and potential harmful effects of new compounds or substances intended for use in the human body [59]. The lower the hemolysis rate, the better the blood compatibility, and if it is less than 5% compared to the positive control exposed to a solution that induces 100% destruction of RBCs, it can be applied as a biomedical material for clinical use [60]. After treating RBCs with the extracts of the prepared sponge composites, RBCs were precipitated by centrifugation, and the supernatant was transparent, as shown in Figure 5a. Additionally, the absorbance at 540 nm of the supernatant was used for determining the Hemolysis Ratio (HR) according to the formula shown in Section 3.8. The HRs were all less than 5% compared to the positive control with red blood cells exposed to distilled water in the study, indicating that the as-prepared composites are blood compatible and can be used as a medical device.

To assess hemostatic capacity, in vitro coagulation capacity was determined. The blood coagulation index (BCI) was expressed as the percentage of absorbance of blood drained from the sponge into PBS compared to the absorbance of a negative control with the same amount of blood added directly to PBS [61]. The more coagulated the blood, the smaller the BCI (Figure 5b) and the clearer the rinse water of the sponge (Figure 5c). This indicates a higher hemostatic potential for GD and GDA compared to G and GA. As shown in Figure 5b, the GD and GDA sponges had a BCI value of less than 10%, which is less than 50% of the value for gelatin sponges. This is because the silanol group and hierarchical porous structures of DB promote blood coagulation [62]. Hydrophilic porous architecture absorbs blood quickly into the diatom frustule, allowing silica and blood to come into contact immediately. Blood clotting requires coagulation factor XII (FXII), which this porous silica surface activates [19]. Therefore, DB imparts hemostatic properties to this sponge composite GDA.

### 2.6. Comparison of Antibiotic Release Patterns and Antimicrobial Activity of Sponge Composites

**Figure 6:**
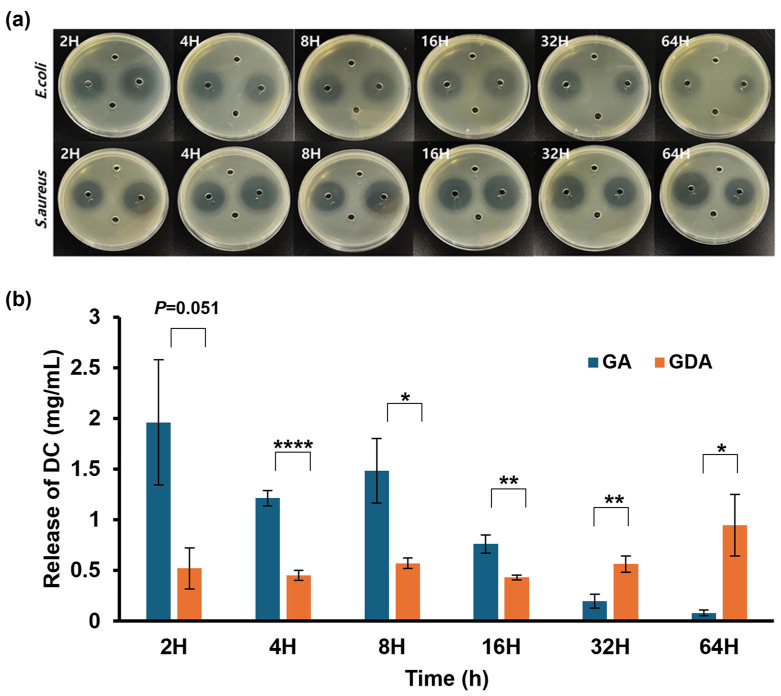
Antibiotic release pattern from sponge composites and their antibacterial activity. (**a**) Antibacterial properties of sponges at 2, 4, 8, 16, 32, and 64 h. Clockwise from the top, the antibacterial abilities of G, GA, GD, and GDA are indicated. (**b**) The amount of DC released at 2, 4, 8, 16, 32, and 64 h. * *p* < 0.05, ** *p* < 0.01 and **** *p* < 0.0001.

Following tooth extraction, a secondary bacterial infection may occur at the extraction site. To this end, the antibacterial efficacy of the sponge was assessed. Figure 6a illustrates the antibacterial capacities of the G, GA, GD, and GDA sponge samples, arranged in a clockwise direction from the top. In the case of GA and GDA, the antibacterial effect was observed to manifest as the loaded DC was released. This observation was made for both *E. coli* and *S. aureus*. As shown in Table 3 and Table 4, the antibacterial ability of GA was dominant from the first 2 to 8 h. At 16 h, similar transparent regions are also observed in GA and GDA. At 32 and 64 h, GDA demonstrated a higher degree of antibacterial activity. No antibacterial activity was observed with G or GD. Next, the amount of DC released from the sponge over time was quantified (Figure 6b). The quantity of antibiotic released from the sponge composite was obtained by converting the size of the growth inhibition zone produced by the extract into a concentration using the equation derived from the linear regression curve. This curve was generated by plotting the sizes of the growth inhibition clear zone obtained on the agar plate (values on the y-axis) as a function of the logarithmic concentration of antibiotic (values on the x-axis), as described in Section 3.5 and Section 3.10. The sponge should be applied to the tooth extraction site until the bleeding ceases. It remains in place for a minimum of three to four days and up to seven days, after which it can be removed once bleeding has stopped [63]. The continuous release of antibiotics ensures consistent treatment and reduces the incidence of adverse effects at the wound site [64]. For GA, a substantial amount of release is observed during the first 2, 4, 8, and 16 h, equivalent to 52% of the initial DC immersion dose of 10 mgmL^−1^, with minimal release observed from hour 32 onwards. On the other hand, in the case of GDA, the delayed release caused by DB leads to a consistent and sustained release of antibiotics. This corresponds to approximately 5% of the initial soaked amount of DC, allowing for uninterrupted delivery of antibiotics to the extraction site. It is likely that the absorption of DC solution occurred in both the gelatinous portion of the sponge and the DB. However, the comparison of release amounts between GA and GDA suggests that drug uptake in GDA is primarily mediated by the DB. Consequently, sponges containing DB initially exhibited reduced antibacterial activity but provided more sustained delayed release than G. This was due to the delayed release of DC from DB [65,66]. The delayed release of antibiotics ensures the continuous delivery of them to the extraction site, thereby preventing the risk of antibiotic resistance [64], while the hemostatic action can effectively initiate the coagulation cascade at the extraction site to prevent complications such as dry socket [63].

### 2.7. Biocompatibility of Sponge Composites

The drug carrier employed for the purpose of delivering a drug to a specific target, as well as the drug itself, is probable to exhibit toxicity towards the cells or animals utilized in the experiment. Hence, the efficacy of the study hinges on the careful choice of a non-toxic and secure drug carrier or drug dosage [67]. The dosage should be antibacterial while being non-toxic to the targeted cells, allowing the cells to remain viable. Therefore, the DC and DC@DB settings for cytotoxicity were determined using a method of diluting the concentration by a factor of two, starting from a concentration of either 50 or 100 µgmL^−1^. This was done based on their MICs and references that show toxicity to human macrophage cells (U 937) at a concentration of 10 µgmL^−1^ [67]. A comparison of the cytotoxicity of free DC and DC immobilized on DB against mammalian cells is shown in Supplementary Figure S2. The Raw264.7 mouse macrophage cell line did not exhibit significant cytotoxicity when exposed to DCs at concentrations below 25 µgmL^−1^. Similarly, DC@DB did not exhibit cytotoxicity at concentrations below 25 µgmL^−1^. At a concentration of 50 µgmL^−1^, cell viability was approximately 80%. In the osteoblast cell line MC3T3 E1, the compounds were not cytotoxic at concentrations below 12.5 µgmL^−1^ and showed 80% cell viability at 25 µgmL^−1^. At a concentration of 50 µgmL^−1^, DC@DB further reduced viability to 75% viability. There was no significant difference in cell viability between the concentrations of free DC and DC@DB.

**Figure 7:**
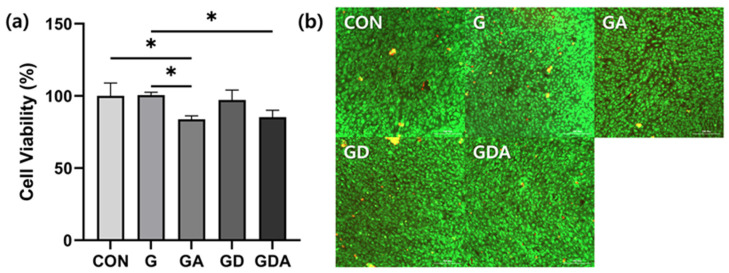
Cytotoxicity in sponge samples. (**a**) Cell viability rate measured through MTS assay. (**b**) Fluorescence microscopy image of MC3T3E1 cells. Live cells were stained green, and dead cells were stained red. * *p* < 0.05.

Next, we investigated the cytotoxicity of composites in MC3T3E1 cells. The cytotoxicity results showed that all the samples were nontoxic, as shown in Figure 7. The cell survival rates of GA and GDA, which were samples containing antibiotics, were 83.9 ± 2.3% and 85.3 ± 4.7%, respectively, confirming that the survival rates were slightly reduced compared to the samples without antibiotics (Figure 7a). Since cytotoxicity testing (ISO 10993-5) considers a decrease in cell viability of more than 30% compared to a negative control to be cytotoxic [68], the composite used in the test showed a decrease in cell viability of less than 20% and is therefore considered biocompatible. The LIVE/DEAD cytotoxicity assay distinguishes between dead and live cells by staining green-fluorescent calcein-AM and red-fluorescent ethidium homodimer-1 [69]. This assay detects cytotoxicity in most eukaryotic cells, with red fluorescence indicating dead cells and green fluorescent cells indicating live cells. The higher the level of cytotoxicity, the greater the proportion of cells that are stained with red fluorescence. In this analysis, green fluorescence was predominantly observed for live cells, with little red color for dead cells (Figure 7b), indicating GDA is biocompatible. Therefore, it was found to be suitable for use as a tooth extraction sponge.

The exploration of diatom biosilica as a drug delivery vehicle could drive further research into biomaterials and their properties, leading to the development of new composites that can deliver various therapeutics, including antibiotics.

## 3. Materials and Methods

### 3.1. Materials

Vancomycin (VM), gentamycin sulfate (GEN), doxycycline hydrochloride (DC), gelatin from bovine skin (type B), glutaraldehyde solution (Grade II, 25% in H_2_O, Sigma-Aldrich, St. Louis, MO, USA), Triton X-100, and lipopolysaccharides (LPS) from *Escherichia coli* O111:B4 were purchased from Sigma-Aldrich (St. Louis, MO, USA). Food-grade diatomite (DE) powder (89% silicon dioxide) was purchased from Wolf Creek Ranch (Kamas, UT, USA). Silicon dioxide (silica, SiO_2_) with an average particle size of 4 μm was purchased from Kojundo Chemical Lab. Co. (Sakado, Japan). CellTiter96^®^AQueous One Solution Cell Proliferation Assay (MTS) was purchased from Promega (Promega, Madison, WI, USA). TNF-α was purchased from PeproTech, ThermoFisher Scientific (Branchburg, NJ, USA). All the other reagents were of analytical grade.

### 3.2. Bacterial Strains

*Escherichia coli* (wild type W2110) and *Staphylococcus aeruginosa* (ATCC 29213) were acquired from Prof. Ha’s Lab in the department of Biotechnology and Bioinformatics at Korea University.

### 3.3. Preparation of Diatom Biosilica

Food-grade diatomaceous earth (DE) was mixed with autoclaved deionized water (DI). The mixture was allowed to settle within 10 min to obtain its intact structure, and the supernatant was removed. The precipitate was repeatedly washed in DI to remove DE debris and dried at 60 °C [70]. After drying, a magnet was used to remove the iron contamination present in the DE. For surface activation, 10 g of iron-removed DE was immersed in a piranha solution, a 3:1 by volume mixture of H_2_SO_4_ and 30% H_2_O_2_ and reacted for 1 h. After treatment, the DE was washed repeatedly with DI until the pH was neutralized, and then dried in a 60 °C oven. From then on, the powder was named diatom biosilica (DB). This process was conducted in the Biological Safety Cabinet (BSC).

### 3.4. Minimum Inhibitory Concentration (MIC) of Antibiotics

As previously reported [71], the antimicrobial activity of antibiotics was tested in the broth microdilution assay. At 37 °C, bacteria were grown to mid-logarithmic phase in Mueller Hinton Broth (MHB). In 96-well plates, 50 µL of antibiotics solution (0–300 µgmL^−1^) from a two-fold serial dilution method was mixed with 3 × 10^6^ CFUmL^−1^ bacterial solution and incubated for overnight at 37 °C at 500 revolutions per minute (rpm). The culture medium was used as a negative control, while bacterial inoculation without antibiotics served as the positive control. The MIC was determined by measuring the lowest antibiotics concentration with the same turbidity (<0.1) as the negative control at 600 nm using a microplate reader (Infinite M200 PRO NanoQuant; TECAN, Männedorf, Switzerland). The assay was conducted three times to ensure accuracy.

### 3.5. Measurement of Loading Efficiency of Antibiotics In Silica Particles (SP) or Diatom Biosilica (DB)

A total of 0.3 mgmL^−1^ of antibiotic sterilized using a sterile syringe filter with a 0.22 μm pore size, was adsorbed to 1 mg of SP or DB, which was sterilized by DI rinsing and UV-radiation. This process was conducted in BSC. The antibiotic supernatant that did not bind to the SPs or DBs was collected. To measure the antibacterial activity using the diffusion susceptibility test, a bacteria-seeded (1.5 × 10^5^ CFUmL^−1^) Mueller Hinton agar plate was used. The Kirby-Bauer disk diffusion susceptibility test [72] was performed at varying antibiotic concentrations using a 10 mgmL^−1^ antibiotic solution to obtain the microbial growth inhibition zone size. A linear regression equation was obtained from a standard curve plotting the size of the generated growth inhibition zone (y-axis, mean diameter in mm) versus the semi-logarithm of the antibiotic amount (x-axis, log10 μgmL^−1^) [73] using Excel (Microsoft Office 365). The aforementioned linear regression equation was then employed to calculate the quantity of antibiotic remaining in the supernatant, based on the size of the growth inhibition zone in the unbound supernatant. The loading efficiency (%) of antibiotics was estimated using the following equations:

$$\mathrm{Adsorption}\;\mathrm{Efficiency}\left(\mathrm{AE}\right)\left(\%\right)=\frac{mass\;of\;bound\;antibiotics}{initial\;mass\;of\;antibiotics\;added}\times 100\;\mathrm{Loading}\;\mathrm{Efficiency}\left(\mathrm{LE}\right)\%=\frac{mass\;of\;bound\;antibiotics}{total\;mass\;of\;delivery\;system}\times 100$$

### 3.6. Fabrication of Gelatin-DB-Antibiotics Composite Sponge (GDA)

Bovine gelatin type B was prepared as a 5% solution by dissolving in DI. DB was mixed at 10 *w*/*v*% in a 5% gelatin solution. For crosslinking, 6.25% glutaraldehyde was added to a final concentration of 0.06% and reacted until crosslinking. When the reaction was complete, it was precipitated in a 100 mM glycine solution for 2 h to remove the remaining cross-linker. Sponges were manufactured by washing with DI, freezing at −20 °C for 12 h, and then freeze-drying. The fabricated sponges were sterilized by UV irradiation for 3 h. The resulting sponge was soaked in DC (10 mgmL^−1^) in the BSC and freeze-dried to obtain the final gelatin-DB-antibiotic (GDA) sponge. The final GDA sponge exhibited a volume of 8.124 ± 0.484 cm^3^ and was found to have a dissimilar cylindrical shape. With 10 mg of DC loaded into approximately 8.124 cm^3^ sponge, there is 1.234 ± 0.074 mg of DC per 1 cm^3^ of sponge volume.

### 3.7. Cytotoxicity

A comparison was made between the cytotoxicity of unbound free DC and DBs containing it (DC@DB) against mouse macrophages (Raw264.7 cells) and MC3T3 E1 mouse preosteoblast. Each well of a 96-well plate was supplemented with the free DC or DC@DB in two-fold serial dilutions, resulting in a final volume of 100 μL. The dilutions were prepared using DMEM (for Raw 264.7) or MEM-α (for MC3T3 E1) medium containing 10% FBS. A concentration of 1 × 10^4^ Raw 264.7 macrophages or 0.5 × 10^4^ MC3T3 E1 cells per 100 μL was introduced into the wells containing the antibiotics, resulting in a final volume of 200 μL. The degree of cell proliferation was assessed using an MTS assay (Promega, USA) performed after either 48 h of incubation or according to the manufacturer’s instructions. Viable cells reduce the MTS tetrazolium compound to produce a colored formazan product. The color intensity at a wavelength of 490 nm is directly proportional to the quantity of viable cells present in the sample. In order to determine cell viability, the absorbance of cells subjected to the antibiotics’ treatment was divided by the absorbance of cells that did not undergo the antibiotics treatment. The final quotient was subsequently converted into a percentage. For testing the cytotoxicity of composite sponges, MC3T3E1 cells were cultured in a CO_2_ incubator for 24 h at 2 × 10^4^ cells per well in a 96-well plate. After incubation, 100 μL of the eluate of sponge composite, obtained by shaking the mixture of 0.4 mL of MEM-α media and 40 mg of composite sponge for 24 h at 37 °C, was added to the cultured cells and incubated for another 24 h. The case of culture in general medium without extract was used as a control. Live/dead cell images were captured using a fluorescence microscope (Cytation^TM^ 7, BioTek, Agilent, Santa Clara, CA, USA) under the same culture conditions to capture the state of the cells using the LIVE/DEAD™ Viability/Cytotoxicity Kit, for mammalian cells according to the manufacturer’s method (ThermoFisher Scientific Korea, Seoul, Republic of Korea).

### 3.8. Hemocompatibility 

The mouse (C57BL/6J 8W/M) blood was used to obtain fresh RBCs through a centrifugation process at 150× *g* for 5 min. The RBCs were then washed three times with PBS. The composite sponge samples were immersed in a saline solution and incubated at 37 °C for 24 h. The RBC stock dispersion (20 μL) was added to 1 mL of the sample extract and incubated for 1 h at 37 °C. After incubation, the mixture was centrifuged at 2000 rpm for 5 min, and the absorbance of the supernatant was measured at 540 nm. The positive control group was mixed with distilled water, and the negative control group was mixed with saline solution. The hemolysis rate was calculated using the following formula [59]:

$$HR\%=\frac{sample\;abs-negative\;control\;abs}{positive\;control\;abs-negative\;control\;abs}\times 100$$

### 3.9. Hemostatic Assay

To prevent thrombosis, 100 μL of mouse (C57BL/6J 8W/M) blood containing 3.8% sodium citrate in a 1:9 ratio was dropped onto a sponge and cross-linked with 10 μL of 0.2 mol/L CaCl_2_ [61]. The samples were incubated at 37 °C and 300 rpm for 30 min, rinsed with 10 mL of PBS, and incubated for an additional 10 min. After this, the sponge in PBS solution was photographed, and the degree of redness was compared. The absorbance of the remaining rinse water (*A*_S_) was measured at 540 nm to evaluate the remaining blood that had not coagulated on the sponge. An aliquot of blood was added directly to the PBS, and the absorbance of the resulting solution was recorded as *A*_0_. The blood clotting index (BCI) was then calculated as *A*_S_/*A*_0_%.

### 3.10. Comparison of Antibiotic Release Patterns and Antimicrobial Activity of Sponge Composites

Before extraction, to minimize the impact on adsorption, the dry sponge with DC was fully immersed in 1 mL of PBS for moisturization and subsequently prepared in a shaking incubator at 37 °C and 200 rpm by adding another 1 mL of PBS. At each subsequent time point, 1 mL of PBS was removed and replaced with an equivalent volume of fresh PBS. The antibacterial activity was measured using the disk diffusion method mentioned in Section 3.5. Wells (4 mm in diameter) were created in an agar plate medium containing 150,000 bacteria per milliliter. Subsequently, time-dependent G, GA, GD, or GDA extracts (50 μL) were added to the wells, and the plates were incubated for at least 12 h. The diameter of the clear zone was then measured to determine the antimicrobial activity of DC released from composites. At each time point, the diameter of the clear zone was converted to DC concentration using a linear regression equation derived by fitting the log-transformed concentrations of DC versus growth inhibition zone size, as outlined in Section 3.5. This allowed us to ascertain the quantity of antibiotic released from the GA and GDA, respectively.

### 3.11. qRT-PCR for mRNA Expression

The mRNA expression was evaluated using quantitative polymerase chain reaction (qPCR) following the previously described protocol [71,74]. The primers used are shown in Supplementary Table S2. To ascertain the alterations in mRNA expression of pertinent genes in response to the addition of DC or DC@DB, respectively, for each condition, the inflammatory response of Raw264.3 cells was induced by the introduction of 100 ngmL^−1^ LPS, while the effects of inflammatory cytokines in MC-3T3 E1 cells were induced by the introduction of TNF-α (10 ngmL^−1^). The expression levels of target genes were quantified by 2^−ΔΔCT^ method [75]. The gene expression data was normalized using GAPDH as a reference gene, as described in a previous study [76].

### 3.12. ATR−FTIR

The peaks of each sponge and material were measured at a resolution of 2 cm^−1^ in the spectral range of 600 to 4000 cm^−1^ through ATR−FT-IR (Perkin Elmer ATR−FT-IR L1600300 Spectrometer Spectrum Two LiTa Lab, Seoul, Republic of Korea). This was performed to check for chemical modifications and bonding.

### 3.13. Statistical Analysis

Statistical analyses were performed using Student’s *t*-test to determine the differences between the two groups. Statistical significance was set at *p* < 0.05.

## 4. Conclusions

When diatom biosilica was used as a carrier for the antibiotic doxycycline, it demonstrated a high adsorption rate of approximately 90% and a loading efficiency of 21%. The complexation of doxycycline with diatom biosilica inhibited the LPS-induced inflammatory response in mouse macrophages by more than 50%. This complex also exhibited a protective effect in osteoblasts against inflammatory cytokines by suppressing the expression of RANKL and DKK-1, while restoring the expression of AMPK and SIRT1.

The gelatin-based diatom-silica composite (GDA) demonstrated biocompatibility, sustained release of antibiotics, and hemostatic effects. Compared to gelatin sponge, which released 52% of antibiotics within the first 16 h, GDA exhibited a sustained release pattern of antibiotics at a constant rate of about 5% over the 64-h measurement period, resulting in a cumulative release of 30%. These findings suggest that GDA, with its sustained delivery of antibiotics and hemostatic properties, holds potential as a material for promoting post-extraction wound healing and managing periodontal disease and peri-implantitis.

However, while diatoms are a natural and sustainable option, extensive research is necessary to evaluate purification methods, immune responses, and unexpected biocompatibility issues to meet the standards required for clinical use. If these limitations are addressed through continued research and innovation, the unique properties and benefits of diatoms could make them a potential candidate for future drug delivery systems.

## Figures and Tables

**Table 1:** Comparison of LE% and AE%.

	Initial Antibiotics (μg)	Adsorbed Antibiotics (μg)	DB Matrix (mg)	LE ^a^ (%)	AE ^b^ (%)
VM@SP	300	53.27 ± 8.70	1	5.05 ± 0.45	17.75 ± 2.88
VM@DB	300	180.08 ± 2.24	1	15.26 ± 0.09	60.03 ± 0.75
GEN@DB	300	271.88 ± 9.38	1	21.38 ± 0.33	90.63 ± 3.13
DC@DB	300	277.29 ± 18.83	1	21.70 ± 0.66	92.43 ± 6.28

^a^ LE is the antibiotic loading efficiency, which is defined as the percentage of the amount of antibiotics in the silica matrix to the total amount of antibiotics combined with the silica matrix. ^b^ AE is the antibiotic adsorption efficiency, defined as the percentage of the amount of antibiotics in the silica matrix to the total amount of antibiotics initially applied to the silica matrix. The value was calculated from three replicate experiments, and values are expressed as the mean ± standard error.

**Table 2:** Minimum Inhibitory Concentration (MIC) of antibiotics.

Antibiotics	*E. coli*	^a^ *p* Value	*S. aureus*	^a^ *p* Value
VM	115.22 ± 51.53		3.75 ± 1.26	
VM@DB	56.25 ± 15.73	0.085	4.89 ± 2.05	0.328
GEN	0.60 ± 0.04		0.48 ± 0.11	
GEN@DB	4.36 ± 0.20	>0.001	2.18 ± 0.10	>0.001
DC	1.48 ± 0.31		0.09 ± 0.02	
DC@DB	7.94 ± 0.54	>0.001	0.34 ± 0.12	0.100

Minimum Inhibitory Concentration (MIC) was calculated in triplicate, and values are expressed as mean ± standard error. The unit is microgram per mL (µgmL^−1^). The value was calculated from three replicate experiments, and values are expressed as the mean ± standard error. ^a^ The *p*-value is calculated by a Student’s *t*-test between the MIC of free form and the MIC of DB combined form against each strain.

**Table 3:** Clear zone size for GA and GDA in *E. coli*.

Sponge Sample	2H	4H	8H	16H	32H	64H
GA	9.83 ± 0.02	8.74 ± 0.06	8.41 ± 1.14	8.03 ± 0.03	5.54 ± 0.32	4.06 ± 0.56
GDA	8.32 ± 0.56	7.53 ± 0.36	8.03 ± 0.24	7.69 ± 0.13	7.70 ± 0.50	7.14 ± 0.53

**Table 4:** Clear zone size for GA and GDA in *S. aureus*.

Sponge Sample	2H	4H	8H	16H	32H	64H
GA	11.66 ± 0.68	12.89 ± 0.35	13.40 ± 0.57	12.41 ± 0.37	10.82 ± 0.48	9.55 ± 0.07
GDA	9.74 ± 0.32	11.51 ± 0.29	11.56 ± 0.32	11.21 ± 0.32	11.87 ± 0.41	11.30 ± 0.23

## Data Availability

The data presented in this study are available in this article.

## Supplementary Materials

The following supporting information can be downloaded at: https://www.mdpi.com/article/10.3390/antibiotics13080714/s1, Table S1: Antibiotics and their characteristic; Table S2: Zeta potential of DB and antibiotics loaded DBs; Table S3: Primers used for qPCR; Figure S1: Anti-inflammatory response of DB in LPS-stimulated RAW264.7 cells; Figure S2: Comparison of cell viability after 2 days of DC or DC@DB treatment.
