# Supplementary material for: Antimicrobial and Hemostatic Diatom Biosilica Composite Sponge
Source: Antibiotics (Basel). 2024 Jul 30;13(8):714. doi: 10.3390/antibiotics13080714 (PMC11350910; doi:10.3390/antibiotics13080714)
Supplement: Supplementary file 1 [file antibiotics-13-00714-s001.zip › antibiotics-3108996-supplementary.pdf]

Supplementary Materials

Antimicrobial and Hemostatic Diatom Biosilica Composite Sponge

Sol Youn [1,†], Mi-Ran Ki [1,2,†], Ki Ha Min[1,2], Mohamed A. A. Abdelhamid[1,3] and Seung Pil Pack [1,*]

1 Department of Biotechnology and Bioinformatics, Korea University, Sejong-Ro 2511, Sejong 30019, Republic of Korea; S. Youn, youseul0419@korea.ac.kr; M.-R.Ki, allheart@korea.ac.kr; M A.A. Abdelhamid, mohamed42@korea.ac.kr; SP Pack, spack@korea.ac.kr

2 Institute of Industrial Technology, Korea University, Sejong-Ro 2511, Sejong 30019, Republic of Korea; M.-R.Ki, allheart@korea.ac.kr, K.H.Min, alsrlgk@korea.ac.kr

3 Department of Botany and Microbiology, Faculty of Science, Minia University, Minia 61519, Egypt; M A.A. Abdelhamid, mohamed42@korea.ac.kr

* Correspondence: spack@korea.ac.kr (S.P.P.); Tel.: +82-44-860-1419 (S.P.P.)

† These authors contributed equally to this work.

Supplementary Table S1. Antibiotics and their characteristics.

Antibiotics	Molecular formular[a]	Molecular weight ($gmol^{-1}$)[a]	p*K*a value	Ref	Physiological charge[b]
Vancomycin	$C_{66}H_{75}Cl_2N_9O_{24}$	1449.2	p*K*a1 = 2.2; p*K*a2 = 7.8; p*K*a3 = 8.9; p*K*a4 = 9.6; p*K*a5 = 10.4; p*K*a6 = 12	[40]	0
Gentamycin sulfate	$C_{60}H_{125}N_{15}O_{25}S$	1488.8	p*K*a1 = 12.55; p*K*a2 = 10.12	[41]	5
Doxycycline HCl	$C_{22}H_{25}ClN_2O_8$	480.9	p*K*a1 = 3.02; p*K*a2 = 7.97; p*K*a3 = 9.15	[43]	0

[a] The molecular formular and molecular weight were obtained from Pubchem (https ://pubchem.ncbi.nlm.nih.gov/) (accessed on 2, June,2024) and [b]the physiological ch arge was obtained from DRUGBANK online (https://go.drugbank.com/drugs) (acces sed on 2, June, 2024).

Supplementary Table S2. Zeta potential of DB and antibiotics loaded DBs

	DB	VM@DB	GEN@DB	DC@DB
Zeta potential (mV)	-30.83 ±0.23	-33.07±0.98	9.63±0.62	-36.32±0.76

DB: diatom biosilica; VM: vancomycin; GEN: gentamycin sulfate; DC: doxycycline·HCl

Supplementary Table S3. Primers used for qPCR

Name	primer	5′ to 3′	Reference
TNF-α	Forward	ATGGCCTCCCTCTCATCAGT	[71]
	Reverse	TGGTTTGCTACGACGTGGG	
IL-1β	Forward	TGCCACCTTTTGACAGTGATG	[71]
	Reverse	AAGGTCCACGGGAAAGACAC	
IL-6	Forward	CTCCCA ACAGACCTGTCTATAC	This study
	Reverse	CCATTGCACAACTCTTTTCTCA	
DKK-1	Forward	CGT GTT TAC AAT GAT GGC TC	This study
	Reverse	GCG TTG GAA TTG ATG AGA AC	
RANKL	Forward	TAA CCT GAT GAA AGG AGG GA	This study
	Reverse	TTG GAC ACC TGA ATG CTA AT	
GAPDH	Forward	CCTGGCCAAGGTCATCCATG	[76]
	Reverse	GCAGGAGACAACCTGGTCCT	

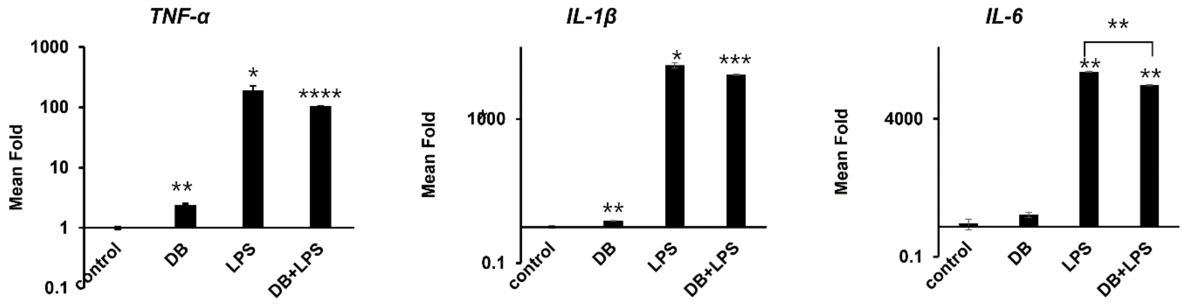

Supplementary Figure S1. Anti-inflammatory response of DB in LPS-stimulated RAW264.7 cells. The mRNA expression levels of inflammatory cytokines, namely TNF-α, IL-1β, and IL-6 were measured by qRT-PCR. Glyceraldehyde-3-Phosphate-Dehydrogenase (GAPDH) gene was used for normalization in gene expression. Gene levels in each treated cell were calculated relative to those in control cells neither LPS nor DB. Values are presented as the mean ± SE (N = 3). **p*<0.05, ***p*<0.01, ****p*<0.001, *****p*<0.0001vs control. ***p*<0.01 vs DB in the presence of LPS.

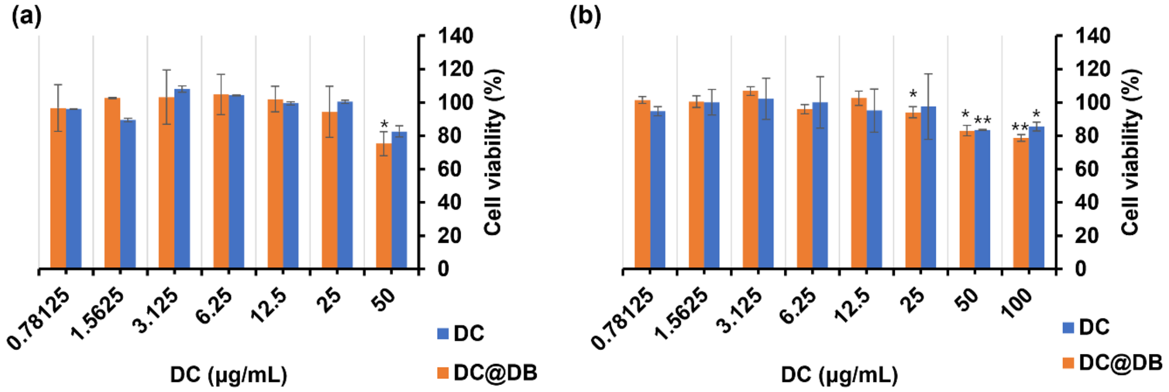

Supplementary Figure S2. Comparison of cell viability after 2 days of DC or DC@DB treatment. The cytotoxicity of DC or DC@DB was measured 2 days after incubation of Raw 264.7 cells (1×10^5 cellsmL^{-1}) (a) or MC3T3 E1 cells (5×10^4 cellsmL^{-1}) (b) in the presence of each indicated concentration of DC. Cell viability was expressed as a percentage of the negative control grown without DC. Values are presented as mean ± SE (N = 3). *p<0.05 or **p<0.01 vs cell viability without DC in each cell.

References in main text

40. Jamzad, S.; Fassihi, R., Role of surfactant and pH on dissolution properties of fenofibrate and glipizide--a technical note. *AAPS PharmSciTech* **2006,** 7, (2), E33.

41. DRUGBANK Gentamycin sulfate. https://go.drugbank.com/salts/DBSALT000690 (June 27, 2024),

43. Shariati, S.; Yamini, Y.; Esrafili, A., Carrier mediated hollow fiber liquid phase microextraction combined with HPLC–UV for preconcentration and determination of some tetracycline antibiotics. *J Chromatogr B* **2009,** 877, (4), 393-400.

71. Ki, M. R.; Kim, S. H.; Park, T. I.; Pack, S. P., Self-Entrapment of Antimicrobial Peptides in Silica Particles for Stable and Effective Antimicrobial Peptide Delivery System. *Int J Mol Sci* **2023,** 24, (22), 16423.

76. Lee, S.; Lee, T. A.; Song, S. J.; Park, T.; Park, B., Hyperproduction of IL-6 caused by aberrant TDP-43 overexpression in high-fat diet-induced obese mice. *FEBS Letters* **2015,** 589, (15), 1825-1831.
